# Supplementary material for: Effect of Resin Infiltration on Enamel: A Systematic Review and Meta-Analysis
Source: J Funct Biomater. 2021 Aug 16;12(3):48. doi: 10.3390/jfb12030048 (PMC8395859; doi:10.3390/jfb12030048)
Supplement: Supplementary file 1 [file jfb-12-00048-s001.zip › jfb-1312444-supplementary.pdf]

Journal of
Functional
Biomaterials

MDPI

Supporting information

Effect of Resin Infiltration on Enamel: A Systematic Review and Meta-analysis

Madalena Soveral [1], Vanessa Machado [1,2,*], João Botelho [1,2], José João Mendes [1] and Cristina Manso [1]

1 Clinical Research Unit (CRU), Centro de Investigação Interdisciplinar Egas Moniz (CiiEM), Egas Moniz—Cooperativa de Ensino Superior, CRL, 2829-511 Almada, Portugal; madalenasvsoveral@gmail.com (M.S.); jbotelho@egasmoniz.edu.pt (J.B.); jmendes@egasmoniz.edu.pt (J.J.M.); mansocristina@gmail.com (C.M.)
2 Evidence-Based Hub, Clinical Research Unit, Centro de Investigação Interdisciplinar Egas Moniz (CiiEM), Egas Moniz Cooperativa de Ensino Superior, CRL, Portugal, 2829-511 Almada, Portugal
* Correspondence: vmachado@egasmoniz.edu.pt

Online Supplemental Information

Summary

Table S1. PRISMA 2009 Checklist………………………………………………………………………………………….…2

Table S2. List of potentially relevant studies not included in the systematic review, along with the reasons for exclusion……..….3

Table S3. Joanna Briggs Institute Clinical Appraisal Checklist for Experimental Studies………………………………………………9

Table S4. Comparison between ROM and SMD results…………………………………………………………………………10

Figure S1. Forest plot of studies evaluating the effect of resin infiltration on the surface roughness of sound enamel…………….10

Figure S2. Forest plot of studies evaluating the effect of resin infiltration on the surface roughness (Ra) of white spot lesions…..10

Figure S3. Forest plot of studies evaluating the effect of resin infiltration on the enamel microhardness of sound enamel………..11

Figure S4. Forest plot of studies evaluating the effect of resin infiltration on the enamel microhardness of white spot lesions……11

Figure S5. Funnel plot and Egger's test for enamel microhardness in sound enamel……………………………………………………12

Figure S6. Funnel plot and Egger's test for enamel microhardness in white spot lesions………………………………………………12

Figure S7. Forest plot of studies evaluating the effect of resin infiltration on the bond strength of sound enamel…………………..12

Figure S8. Forest plot of studies evaluating the effect of resin infiltration on the bond strength of white spot lesions………………13

Figure S9. Forest plot of studies evaluating the penetration depth of infiltration on caries lesions according to time of application of infiltrant (in minutes)………13

Figure S10. Funnel plot and Egger's test for penetration depth……………………………………………………………………….14

Table S1. PRISMA 2009 Checklist.

Section/Topic	#	Checklist item	Reported on page #
		**TITLE**	
Title	1	Identify the report as a systematic review, meta-analysis, or both.	1
		**ABSTRACT**	
Structured summary	2	Provide a structured summary including, as applicable: background; objectives; data sources; study eligibility criteria, participants, and interventions; study appraisal and synthesis methods; results; limitations; conclusions and implications of key findings; systematic review registration number.	1
		**INTRODUCTION**	
Rationale	3	Describe the rationale for the review in the context of what is already known.	1–2
Objectives	4	Provide an explicit statement of questions being addressed with reference to participants, interventions, comparisons, outcomes, and study design (PICOS).	1–2
		**METHODS**	
Protocol and registration	5	Indicate if a review protocol exists, if and where it can be accessed (e.g., Web address), and, if available, provide registration information including registration number.	2
Eligibility criteria	6	Specify study characteristics (e.g., PICOS, length of follow-up) and report characteristics (e.g., years considered, language, publication status) used as criteria for eligibility, giving rationale.	2
Information sources	7	Describe all information sources (e.g., databases with dates of coverage, contact with study authors to identify additional studies) in the search and date last searched.	2
Search	8	Present full electronic search strategy for at least one database, including any limits used, such that it could be repeated.	2–3
Study selection	9	State the process for selecting studies (i.e., screening, eligibility, included in systematic review, and, if applicable, included in the meta-analysis).	2–3
Data collection process	10	Describe method of data extraction from reports (e.g., piloted forms, independently, in duplicate) and any processes for obtaining and confirming data from investigators.	3
Data items	11	List and define all variables for which data were sought (e.g., PICOS, funding sources) and any assumptions and simplifications made.	2–3
Risk of bias in individual studies	12	Describe methods used for assessing risk of bias of individual studies (including specification of whether this was done at the study or outcome level), and how this information is to be used in any data synthesis.	3
Summary measures	13	State the principal summary measures (e.g., risk ratio, difference in means).	3–4
Synthesis of results	14	Describe the methods of handling data and combining results of studies, if done, including measures of consistency (e.g., I^2) for each meta-analysis.	3–4
Risk of bias across studies	15	Specify any assessment of risk of bias that may affect the cumulative evidence (e.g., publication bias, selective reporting within studies).	NA
Additional analyses	16	Describe methods of additional analyses (e.g., sensitivity or subgroup analyses, meta-regression), if done, indicating which were pre-specified.	3–4
		**RESULTS**	
Study selection	17	Give numbers of studies screened, assessed for eligibility, and included in the review, with reasons for exclusions at each stage, ideally with a flow diagram.	4
Study characteristics	18	For each study, present characteristics for which data were extracted (e.g., study size, PICOS, follow-up period) and provide the citations.	4–9
Risk of bias within studies	19	Present data on risk of bias of each study and, if available, any outcome level assessment (see item 12).	10
Results of individual studies	20	For all outcomes considered (benefits or harms), present, for each study: (a) simple summary data for each intervention group (b) effect estimates and confidence intervals, ideally with a forest plot.	10–14
Synthesis of results	21	Present results of each meta-analysis done, including confidence intervals and measures of consistency.	10–14

Risk of bias across studies	22	Present results of any assessment of risk of bias across studies (see Item 15).	NA
Additional analysis	23	Give results of additional analyses, if done (e.g., sensitivity or subgroup analyses, meta-regression [see Item 16]).	12–14
		**DISCUSSION**	
Summary of evidence	24	Summary of evidence	24
Limitations	25	Limitations	25
Conclusions	26	Conclusions	26
		**FUNDING**	
Funding	27	Funding	27

NA–Not applicable.
From: Moher D, Liberati A, Tetzlaff J, Altman DG, The PRISMA Group (2009). Preferred Reporting Items for Systematic Reviews and Meta-Analyses: The PRISMA Statement. PLoS Med 6(7): e1000097. doi:10.1371/journal.pmed1000097.
For more information, visit: www.prisma-statement.org.(accessed on 15th May 2021).

Table S2. List of potentially relevant studies not included in the systematic review, along with the reasons for exclusion.

N	Year	Reference	Exclusion reason
1	1976	Robinson C, Hallsworth AS, Weatherell JA, Künzel W. Arrest and Control of Carious Lesions: A Study Based on Preliminary Experiments with Resorcinol-Formaldehyde Resin. Journal of Dental Research. 1976;55(5):812-818. doi:10.1177/00220345760550051601	Data presented in thresholds
2	2009	Kugel G, Arsenault P, Papas A. Treatment modalities for caries management, including a new resin infiltration system. Compend Contin Educ Dent. 2009 Oct;30 Spec No 3:1-10; quiz 11-2. PMID: 19894293.	Unable to access
3	2010	Mueller J1, Yang F, Neumann K, K. A. (n.d.). Effects of Different Finishing Procedures and Materials on Surface Roughness of Infiltrated Subsurface Bovine Enamel Lesions. Quintessence Int 42, 42(2):, 135–147.	Thesis
4	2011	Mueller J, Yang F, Neumann K, Kielbassa AM. Surface tridimensional topography analysis of materials and finishing procedures after resinous infiltration of subsurface bovine enamel lesions. Quintessence Int. 2011 Feb;42(2):135-47. PMID: 21359248.	Unable to access
5	2011	Wiegand A, Stawarczyk B, Kolakovic M, Hämmerle CH, Attin T, Schmidlin PR. Adhesive performance of a caries infiltrant on sound and demineralised enamel. J Dent. 2011 Feb;39(2):117-21. doi: 10.1016/j.jdent.2010.10.010. Epub 2010 Oct 17. PMID: 20959133.	Unable to access
6	2011	Bidarkar, Atul. "In vitro prevention of secondary demineralization by icon (infiltration concept)." MS (Master of Science) thesis, University of Iowa, 2011. https://doi.org/10.17077/etd.1u6479b8_.(accessed on 4th May 2021).	Thesis
7	2012	Naidu E, Stawarczyk B, Tawakoli PN, Attin R, Attin T, Wiegand A. Shear bond strength of orthodontic resins after caries infiltrant preconditioning. Angle Orthod. 2013 Mar;83(2):306-12. doi: 10.2319/052112-409.1. Epub 2012 Aug 22. PMID: 22908947.	Data presented in thresholds
8	2012	Jia L, Stawarczyk B, Schmidlin PR, Attin T, Wiegand A. Effect of caries infiltrant application on shear bond strength of different adhesive systems to sound and demineralized enamel. J Adhes Dent. 2012 Dec;14(6):569-74. doi: 10.3290/j.jad.a25685. PMID: 22724105.	Incomplete data
9	2012	Yang F, Mueller J, Kielbassa AM. Surface substance loss of subsurface bovine enamel lesions after different steps of the resinous infiltration technique: a 3D topography analysis. Odontology. 2012 Jul;100(2):172-80. doi: 10.1007/s10266-011-0031-4. Epub 2011 Jun 16. PMID: 21678019.	Incomplete data
10	2012	Bailey, Melissa Wu, "Effectiveness of Resin Infiltration and Mi Paste Cpp-Acp in Masking White Spot Lesions" (2012). Loma Linda University Electronic Theses, Dissertations & Projects. 72. http://scholarsrepository.llu.edu/etd/72. (accessed on 4th May 2021)	Incomplete data
11	2012	Lincoln, T. A. (2012). The Surface Properties of Teeth Treated with Resin Infiltration or Amorphous Calcium Phosphate. May.	Thesis

12	2012	Hanashiro, F. S. (2012). Avaliação in vitro das superfícies vestibulares desmineralizadas e restauradas com resina infiltrante [Universidade de São Paulo]. https://doi.org/10.11606/T.23.2012.tde-13042013-120154. (accessed on 4th May 2021)	Thesis
13	2012	De Araújo, Larissa Sgarbosa Napoleão; Gaglianone, Lívia Aguilera; Marchi, Giselle Maria; Aguiar, Flávio Henrique Baggio; Araújo, Giovana Spagnolo Albamonte; Puppin-Rontani, R. M. (2012). Tratamento de lesão cariosa proximal através da infiltração com resina de baixa viscosidade. Revista Dental Press de Estética . Jan-Mar2012, Vol. 9 Issue 1, P76-84. 9p. 13 Color Photographs, 4 Black and White Photographs.	Unable to acess
14	2013	Araújo GS, Sfalcin RA, Araújo TG, Alonso RC, Puppin-Rontani RM. Evaluation of polymerization characteristics and penetration into enamel caries lesions of experimental infiltrants. J Dent. 2013 Nov;41(11):1014-9. doi: 10.1016/j.jdent.2013.08.019. Epub 2013 Sep 1. PMID: 24004967.	Incomplete data
15	2013	Yuan H, Li J, Chen L, Cheng L, Cannon RD, Mei L. Esthetic comparison of white-spot lesion treatment modalities using spectrometry and fluorescence. Angle Orthod. 2014 Mar;84(2):343-9. doi: 10.2319/032113-232.1. Epub 2013 Aug 28. PMID: 23984991.	Data presented in thresholds
16	2013	Taher NM. Atomic force microscopy and tridimensional topography analysis of human enamel after resinous infiltration and storage in water. Saudi Med J. 2013 Apr;34(4):408-14. Erratum in: Saudi Med J. 2015 Jul;36(7):885. PMID: 23552595.	Unsuitable control group
17	2013	Yetkiner, E., Özcan, M., Wegehaupt, F. J., Wiegand, A., Eden, E., & Attin, T. (2013). Effect of a low-viscosity adhesive resin on the adhesion of metal brackets to enamel etched with hydrochloric or phosphoric acid combined with conventional adhesives. The Journal of Adhesive Dentistry, 15(6), 575–57581. https://doi.org/10.3290/j.jad.a29607. (accessed on 4th May 2021)	Unsuitable control group
18	2014	Tostes MA, Santos E Jr, Camargo SA Jr. Effect of resin infiltration on the nanomechanical properties of demineralized bovine enamel. Indian J Dent. 2014 Jul;5(3):116-22. doi: 10.4103/0975-962X.140819. PMID: 25565739; PMCID: PMC4213871.	Data presented in thresholds
19	2014	Borges AB. The concept of resin infiltration technique and its multiple applications. J Contemp Dent Pract. 2014 May 1;15(3):i. PMID: 25307827.	Unable to access
20	2014	Resin infiltration effects in a caries-active environment. J Calif Dent Assoc. 2014 Jun;42(6):372. PMID: 25080756.	Unable to access
21	2014	Subramaniam P, Girish Babu KL, Lakhotia D. Evaluation of penetration depth of a commercially available resin infiltrate into artificially created enamel lesions: An in vitro study. J Conserv Dent. 2014 Mar;17(2):146-9. doi: 10.4103/0972-0707.128054. PMID: 24778511; PMCID: PMC4001271.	Incomplete data
22	2014	Chay PL, Manton DJ, Palamara JE. The effect of resin infiltration and oxidative pre-treatment on microshear bond strength of resin composite to hypomineralised enamel. Int J Paediatr Dent. 2014 Jul;24(4):252-67. doi: 10.1111/ipd.12069. Epub 2013 Oct 17. PMID: 24134408.	Unsuitable control group
23	2014	Crombie F, Manton D, Palamara J, Reynolds E. Resin infiltration of developmentally hypomineralised enamel. Int J Paediatr Dent. 2014 Jan;24(1):51-5. doi: 10.1111/ipd.12025. Epub 2013 Feb 15. PMID: 23410530.	Unsuitable control group
24	2014	Ou XY, Zhao YH, Ci XK, Zeng LW. Masking white spots of enamel in caries lesions with a non-invasive infiltration technique in vitro. Genet Mol Res. 2014 Aug 29;13(3):6912-9. doi: 10.4238/2014.August.29.14. PMID: 25177972.	Incomplete data
25	2014	Arnold WH, Bachstaedter L, Benz K, Naumova EA. Resin infiltration into differentially extended experimental carious lesions. Open Dent J. 2014;8:251-256. Published 2014 Dec 29. doi:10.2174/1874210601408010251	Extension of the lesions- Not Enought Studies
26	2014	Alexandru Ogodescu, Adrian Manescu, Ana Emilia Ogodescu, Alessandra Giuliani, Carmen Todea, "Micro-CT application for infiltration technology in paedodontics and orthodontics," Proc. SPIE 8925, Fifth International Conference on Lasers in Medicine: Biotechnologies Integrated in Daily Medicine, 892508 (14 January 2014); https://doi.org/10.1117/12.2044116. (accessed on 4th May 2021)	Data presented in thresholds
27	2014	Munhoz, T. (2014). Effect of bonding protocol on shear bond strength of orthodontic brackets: An in vitro study. Revista Materia, 19(3), 212–217. https://doi.org/10.1590/S1517-70762014000300004. (accessed on 4th May 2021)	Unsuitable control group

28	2014	MACIEL, Patricia Pereira. Avaliação da hibridização do esmalte dentário através de fluxo eletrocinético. 2014. 84 f. Dissertação (Mestrado em Odontologia) - Universidade Federal da Paraíba, João Pessoa, 2014.	Thesis
29	2014	Pérez, R., Quijada, V., & Uribe, S. (2014). Revista Clínica de Periodoncia , Implantología y Rehabilitación Oral Confocal laser microscopy analysis of resin infiltration in fluorotic teeth. 7(2), 53–58.	Unsuitable control group
30	2015	Montasser MA, El-Wassefy NA, Taha M. In vitro study of the potential protection of sound enamel against demineralization. Prog Orthod. 2015;16:12. doi: 10.1186/s40510-015-0080-2. Epub 2015 May 22. PMID: 26061985; PMCID: PMC4440871.	Data presented in thresholds
31	2015	Natarajan AK, Fraser SJ, Swain MV, Drummond BK, Gordon KC. Raman spectroscopic characterisation of resin-infiltrated hypomineralised enamel. Anal Bioanal Chem. 2015 Jul;407(19):5661-71. doi: 10.1007/s00216-015-8742-y. Epub 2015 May 13. PMID: 25967150.	Data presented in thresholds
32	2015	Skucha-Nowak M. Attempt to assess the infiltration of enamel made with experimental preparation using a scanning electron microscope. Open Med (Wars). 2015 Apr 3;10(1):238-248. doi: 10.1515/med-2015-0036. PMID: 28352701; PMCID: PMC5152982.	Data presented in thresholds
33	2015	Askar H, Lausch J, Dörfer CE, Meyer-Lueckel H, Paris S. Penetration of micro-filled infiltrant resins into artificial caries lesions. J Dent. 2015 Jul;43(7):832-8. doi: 10.1016/j.jdent.2015.03.002. Epub 2015 Mar 10. PMID: 25769265.	Unsuitable control group
34	2015	Ulrich I, Mueller J, Wolgin M, Frank W, Kielbassa AM. Tridimensional surface roughness analysis after resin infiltration of (deproteinized) natural subsurface carious lesions. Clin Oral Investig. 2015 Jul;19(6):1473-83. doi: 10.1007/s00784-014-1372-5. Epub 2014 Dec 9. PMID: 25483122.	Different type of analyze
35	2015	Mews L, Kern M, Ciesielski R, Fischer-Brandies H, Koos B. Shear bond strength of orthodontic brackets to enamel after application of a caries infiltrant. Angle Orthod. 2015 Jul;85(4):645-50. doi: 10.2319/013014-82.1. Epub 2014 Aug 26. PMID: 25157972.	Unsuitable control group
36	2015	Mugisa, I. (2015). Microhardness and caries resistance of an infiltrant resin in a novel artificial mouth. August 2010.	Thesis
37	2015	Gabrielle, T., & Ara, F. (2015). "Influência de solventes nas propriedades físico -químicas de infiltrantes resinosos experimentais" " Influence of solvents on the physicochemical properties of experimental resin infiltrants."	Thesis
38	2015	Freitas, M. C. C. de A. (2015). Efeito de um infiltrante resinoso no tratamento de lesões de mancha branca: análise in vitro e in situ [Universidade de São Paulo]. https://doi.org/10.11606/T.25.2015.tde-28102015-092917. (accessed on 4th May 2021)	Thesis
39	2015	Tereza, G. P. G. (2015). Influência da remoção do excesso de materiais adesivos sobre o esmalte erodido, na resistência a desafio erosivo in vitro [Universidade de São Paulo]. https://doi.org/10.11606/D.25.2015.tde-25112015-094345. (accessed on 4th May 2021)	Thesis
40	2016	Farias de Lacerda AJ, Ferreira Zanatta R, Crispim B, Borges AB, Gomes Torres CR, Tay FR, Pucci CR. Influence of de/remineralization of enamel on the tensile bond strength of etch-and-rinse and self-etching adhesives. Am J Dent. 2016 Oct;29(5):289-293. PMID: 29178743.	Unable to access
41	2016	Skucha-Nowak M, Machorowska-Pieniążek A, Tanasiewicz M. Assessing the Penetrating Abilities of Experimental Preparation with Dental Infiltrant Features Using Optical Microscope: Preliminary Study. Adv Clin Exp Med. 2016 Sep-Oct;25(5):961-969. doi: 10.17219/acem/63007. PMID: 28028962.	Incomplete data
42	2016	Inagaki LT, Alonso RC, Araújo GA, de Souza-Junior EJ, Anibal PC, Höfling JF, Pascon FM, Puppin-Rontani RM. Effect of monomer blend and chlorhexidine-adding on physical, mechanical and biological properties of experimental infiltrants. Dent Mater. 2016 Dec;32(12):e307-e313. doi: 10.1016/j.dental.2016.09.028. Epub 2016 Oct 28. PMID: 28327302.	Unsuitable control group
43	2016	Ionta, F. Q., Boteon, A. P., Moretto, M. J., Júnior, O. B., Honório, H. M., Silva, T. C., Wang, L., & Rios, D. (2016). Penetration of resin-based materials into initial erosion lesion: A confocal microscopic study. Microscopy Research and Technique, 79(2), 72–80. https://doi.org/10.1002/jemt.22607. (accessed on 4th May 2021)	Unsuitable control group
44	2016	Al-Dabagh, D. J. N., & Balasim, M. (2016). The Influence of Caries Infiltrant Combined with and without Conventional Adhesives on Sealing of Sound Enamel : In Vitro Study. Journal of	Unable to access

		Baghdad College of Dentistry, 28(2), 119–125. https://doi.org/10.12816/0028233. (accessed on 4th May 2021)	
45	2016	Commander, L., Corps, D., States, U., & Health, P. (2016). كتابة.Pdf. June.	Thesis
46	2016	Dos Reis, B. C., Lacerda, A. J. F. de, Canepele, T. M. F., Borges, A. B., Yui, K. C. K., Torres, C. R. G., & Pucci, C. R. (2016). Evaluation of bond strength of composite resin to enamel demineralized, exposed to remineralization and subjected to caries infiltration. Brazilian Dental Science, 19(1), 48. https://doi.org/10.14295/bds.2016.v19i1.1212. (accessed on 4th May 2021)	Unsuitable control group
47	2016	Vianna, Julia Sotero; Marquezan, Mariana; Thiago Chon Leon Lau; San'Anna, E. F. (2016). Colagem de braquetes em lesões de mancha branca pré-tratadas por meio de dois métodos. Dental Press Journal of Orthodontics. Mar/Apr2016, Vol. 21 Issue 2, P39-44. 6p.	Unable to acess
48	2016	Elhiny, O. A., Elattar, H. S., & Salem, G. A. (2016). CODEN (USA): PCHHAX. 8(18), 100–106.	Unsuitable control group
49	2016	Elhiny, O., & Salem, G. (2016). Will Resin Infiltration With Icon Prevent Enamel Demineralization Around Orthodontic Brackets? International Journal of Advanced Research, 4(9), 1661–1667. https://doi.org/10.21474/ijar01/1626. (accessed on 4th May 2021)	Unsuitable control group
50	2016	Arnold WH, Meyer AK, Naumova EA. Surface Roughness of Initial Enamel Caries Lesions in Human Teeth After Resin Infiltration. Open Dent J. 2016;10:505-515. Published 2016 Sep 23. doi:10.2174/1874210601610010505	Different type of analyze
51	2017	Piątek-Jakubek K, Nowak J, Bołtacz-Rzepkowska E. Influence of infiltration technique and selected demineralization methods on the roughness of demineralized enamel: An in vitro study. Adv Clin Exp Med. 2017 Nov;26(8):1179-1188. doi: 10.17219/acem/66209. PMID: 29264873.	Unsuitable control group
52	2017	Swamy DF, Barretto ES, Mallikarjun SB, Dessai SSR. In vitro Evaluation of Resin Infiltrant Penetration into White Spot Lesions of Deciduous Molars. J Clin Diagn Res. 2017 Sep;11(9):ZC71-ZC74. doi: 10.7860/JCDR/2017/28146.10599. Epub 2017 Sep 1. PMID: 29207838; PMCID: PMC5713860.	Unsuitable control group
53	2017	Schneider H, Park KJ, Rueger C, Ziebolz D, Krause F, Haak R. Imaging resin infiltration into non-cavitated carious lesions by optical coherence tomography. J Dent. 2017 May;60:94-98. doi: 10.1016/j.jdent.2017.03.004. Epub 2017 Mar 10. PMID: 28286174.	Data presented in thresholds
54	2017	Lausch J, Askar H, Paris S, Meyer-Lueckel H. Micro-filled resin infiltration of fissure caries lesions in vitro. J Dent. 2017 Feb;57:73-76. doi: 10.1016/j.jdent.2016.12.010. Epub 2016 Dec 30. PMID: 28043846.	Incomplete data
55	2017	Sfalcin RA, Correr AB, Morbidelli LR, Araújo TGF, Feitosa VP, Correr-Sobrinho L, Watson TF, Sauro S. Influence of bioactive particles on the chemical-mechanical properties of experimental enamel resin infiltrants. Clin Oral Investig. 2017 Jul;21(6):2143-2151. doi: 10.1007/s00784-016-2005-y. Epub 2016 Nov 12. PMID: 27838844.	Unsuitable control group
56	2017	Zamorano, X., Valenzuela, V., Daniels, A. and Iturain, A. (2017) SEM Comparison of Penetration in Artificial White Spots Lesion between an Infiltrant Resin and Two Adhesive Systems. Open Journal of Stomatology, 7, 147-157. https://doi.org/10.4236/ojst.2017.73010. (accessed on 4th May 2021)	Unsuitable control group
57	2017	Easterly, D. E. (2017). An Investigation of Surface Characteristics of Enamel Treated An Investigation of Surface Characteristics of Enamel Treated with Infiltrative Resin: A Scanning Electron Microscopy Study with Infiltrative Resin: A Scanning Electron Microscopy Study. https://scholarscompass.vcu.edu/etd. (accessed on 4th May 2021)	Thesis
58	2017	RIBEIRO, Mariana Dias Flor. Avaliação das propriedades físicas e antibacterianas de infiltrantes experimentais contendo sal de iodônio e quitosana. 2017. 1 recurso online (56 p.). Dissertação (mestrado) - Universidade Estadual de Campinas, Faculdade de Odontologia de Piracicaba, Piracicaba, SP. Disponível em: <http://www.repositorio.unicamp.br/handle/REPOSIP/331395>. Acesso em: 1 set. 2018. (accessed on 4th May 2021)	Thesis
59	2017	SOUZA, Caroline Mathias Carvalho de. Influência da incorporação de um sal de ônio sobre propriedades físicas de infiltrantes experimentais contendo diferentes diluentes. 2017. 1 recurso online (61 p.). Dissertação (mestrado) - Universidade Estadual de Campinas, Faculdade de Odontologia de Piracicaba, Piracicaba, SP. Disponível em:	Thesis

		<http://www.repositorio.unicamp.br/handle/REPOSIP/331942>. Acesso em: 1 set. 2018. (accessed on 4th May 2021).	
60	2017	Hariyati, 2017. (2017). Innovative Approaches to Quality Assurance in Healthcare. Roszdravnadzor Bulletin, 6, 5–9.	Thesis
61	2018	Freitas MCCA, Nunes LV, Comar LP, Rios D, Magalhães AC, Honório HM, Wang L. In vitro effect of a resin infiltrant on different artificial caries-like enamel lesions. Arch Oral Biol. 2018 Nov;95:118-124. doi: 10.1016/j.archoralbio.2018.07.011. Epub 2018 Aug 6. PMID: 30099240.	Unsuitable control group
62	2018	Abbas BA, Marzouk ES, Zaher AR. Treatment of various degrees of white spot lesions using resin infiltration-in vitro study. Prog Orthod. 2018 Aug 6;19(1):27. doi: 10.1186/s40510-018-0223-3. PMID: 30079435; PMCID: PMC6081872.	Unsuitable control group
63	2018	Alizae Marny Mohamed, Kiong Hung Wong, Wan Jen Lee, Murshida Marizan Nor, Haizal Mohd Hussaini, Tanti Irawati Rosli, In vitro study of white spot lesion: Maxilla and mandibular teeth, The Saudi Dental Journal, Volume 30, Issue 2, 2018, Pages 142-150, ISSN 1013-9052, https://doi.org/10.1016/j.sdentj.2017.12.001. (accessed on 4th May 2021).	Unsuitable control group
64	2018	Yazkan B, Ermis RB. Effect of resin infiltration and microabrasion on the microhardness, surface roughness and morphology of incipient carious lesions. Acta Odontol Scand. 2018 Oct;76(7):473-481. doi: 10.1080/00016357.2018.1437217. Epub 2018 Feb 15. PMID: 29447057.	Unsuitable control group
65	2018	Wisam W. Alhamadi et al. (2018), Effect of Resin Infiltrant Pretreatment on Shear Bond Strength of Metal Orthodontic Brackets in Vitro Study. Int J Dent & Oral Heal. 4:7, 105-110.	Unsuitable control group
66	2018	Yadak, A., EL-Sayed, H., & Genaid, T. (2018). Micro-Leakage and Penetration of a Resin Infiltrant Versus Two Conventional Fissure Sealants in Induced Occlusal Fissure. Researchgate.Net, December.	Data presented in thresholds
67	2018	Emanuela, J., & Dos, D. (2018). Universidade Estadual De Campinas Janaina Emanuela Damasceno Dos Santos Avaliação Das Propriedades Físico-Químicas E Da Profundidade De Penetração De Infiltrantes Avaliação Das Propriedades Físico-Químicas E Da.	Thesis
68	2018	Jorne, A. (2018). Instituto universitário egas moniz. 6–7.	Thesis
69	2018	Nassar, A. A. M. (2018). Effect of three different esthetic treatments on the microardness and surface roughness of (ICON) treated teeth. Al-Azhar Journal of Dental Science, 21(5), 539–543. https://doi.org/10.21608/ajdsm.2018.71698. (accessed on 4th May 2021)	Data presented in thresholds
70	2018	Attia, R. (2018). Effect of resin infiltrant and fluoride varnish on micro-hardness of de-mineralized enamel submitted to pH challenge. Egyptian Dental Journal, 64(1), 499–508. https://doi.org/10.21608/edj.2018.78053. (accessed on 4th May 2021)	Values present in other article
71	2019	Torres CRG, Zanatta RF, Fonseca BM, Borges AB. Fluorescence properties of demineralized enamel after resin infiltration and dental bleaching. Am J Dent. 2019 Feb;32(1):43-46. PMID: 30834731.	Unable to acess
72	2019	Wu L, Geng K, Gao Q. Effects of different anti-caries agents on microhardness and superficial microstructure of irradiated permanent dentin: an in vitro study. BMC Oral Health. 2019 Jun 14;19(1):113. doi: 10.1186/s12903-019-0815-4. PMID: 31200708; PMCID: PMC6570839.	Unsuitable control group
73	2019	Al Tuwirqi AA, Alshammari AM, Felemban OM, Ali Farsi NM. Comparison of Penetration Depth and Microleakage of Resin Infiltrant and Conventional Sealant in Pits and Fissures of Permanent Teeth In Vitro. J Contemp Dent Pract. 2019 Nov 1;20(11):1339-1344. PMID: 31892688.	Unable to access
74	2019	Chen M, Li JZ, Zuo QL, Liu C, Jiang H, Du MQ. Accelerated aging effects on color, microhardness and microstructure of ICON resin infiltration. Eur Rev Med Pharmacol Sci. 2019 Sep;23(18):7722-7731. doi: 10.26355/eurrev_201909_18981. PMID: 31599398.	Unsuitable control group
75	2019	Yadav P, Desai H, Patel K, Patel N, Iyengar S. A comparative quantitative & qualitative assessment in orthodontic treatment of white spot lesion treated with 3 different commercially available materials - In vitro study. J Clin Exp Dent. 2019 Sep 1;11(9):e776-e782. doi: 10.4317/jced.56044. PMID: 31636868; PMCID: PMC6797449.	Data presented in thresholds
76	2019	Aswani R, Chandrappa V, Uloopi KS, Chandrasekhar R, RojaRamya KS. Resin Infiltration of Artificial Enamel Lesions: Evaluation of Penetration Depth, Surface Roughness and Color Stability. Int J Clin Pediatr Dent. 2019 Nov-Dec;12(6):520-523. doi: 10.5005/jp-journals-10005-1692. PMID: 32440067; PMCID: PMC7229383.	Unsuitable control group

77	2019	Arora TC, Arora D, Tripathi AM, Yadav G, Saha S, Dhinsa K. An In-Vitro evaluation of resin infiltration system and conventional pit and fissure sealant on enamel properties in white spot lesions. J Indian Soc Pedod Prev Dent 2019;37:133-9	Unsuitable control group
78	2019	Zhu, C., Chen, L., Ou, L., Geng, Q., Jiang, W., Lv, X., Wu, X., Ci, H., Liu, Q., Yao, Y., Pentadbiran, P., Persekutuan, K., Kami, R., Ketua, S., Kementerian, S., Persekutuan, J., Pentadbiran, S., Kerajaan, S., Berkanun, B., … Flynn, D. (2019). Communication Process for Farmers' Adoption of Sufficiency Economy Philosophy in Chiang Mai Province. 8(2), 2019. https://doi.org/10.22201/fq.18708404e.2004.3.66178. (accessed on 4th May 2021)	Thesis
79	2019	RUSSI, Tereza Maria Amorim Zaranza de Carvalho. Avaliação Microbiológica da Superfície de Infiltrante Resinoso Submetido a Diferentes Sistemas de Polimento. 2019.Dissertação (Mestrado Acadêmico em Ciências Odontológicas) - Centro Universitário Christus, Fortaleza, 2019.	Thesis
80	2019	PEDREIRA, Priscila Regis Matos. Influência da incorporação de óxido de bário e zircônia nas propriedades físico-químicas de infiltrantes experimentais e comercial. 2019. 1 recurso online (66 p.). Dissertação (mestrado) - Universidade Estadual de Campinas, Faculdade de Odontologia de Piracicaba, Piracicaba, SP.	Thesis
81	2019	Ozyurt, E., Arisu, H. D., & Turkoz, E. (2019). In vitro comparison of the effectiveness of a resin infiltration system and a dental adhesive system in dentinal tubule penetration Dentinal Penetration of Dental Resins. Clinical and Experimental Health Sciences, 36290600. https://doi.org/10.33808/clinexphealthsci.599847. (accessed on 4th May 2021)	Dentin tubule penetration- Not Enought Studies
82	2019	Abd Alhady, A., & Mohamed, H. (2019). Evaluation of the thickness and depth of penetration of icon into the artificial enamel white spot lesion. Egyptian Dental Journal, 65(4), 3795–3803. https://doi.org/10.21608/edj.2019.76028. (accessed on 4th May 2021)	Data presented in thresholds
83	2020	Fahmy, R. S., Maher, K., & Refai, W. (2020). Effect of resin infiltration concept on bonding strength of effect of resin infiltration concept on bonding Ragi Samy Fahmy *, Kareem Maher Mohamed ** and Wael Mohamed Mobarak Refai ***. October.	Unsuitable control group
84	2020	Simunovic Anicic, M.; Goracci, C.; Juloski, J.; Miletic, I.; Mestrovic, S. The Influence of Resin Infiltration Pretreatment on Orthodontic Bonding to Demineralized Human Enamel. Appl. Sci. 2020, 10, 3619. https://doi.org/10.3390/app10103619. (accessed on 4th May 2021)	Unsuitable control group
85	2020	Kielbassa AM, Leimer MR, Hartmann J, Harm S, Pasztorek M, Ulrich IB (2020) Ex vivo investigation on internal tunnel approach/internal resin infiltration and external nanosilver-modified resin infiltration of proximal caries exceeding into dentin. PLoS ONE 15(1): e0228249. https://doi.org/10.1371/journal.pone.0228249. (accessed on 4th May 2021).	Data presented in thresholds
86	2020	Wu L, Geng K, Gao Q. Effects of different anti-caries procedures on microhardness and micromorphology of irradiated permanent enamel. Dent Mater J. 2020 Jan 31;39(1):118-125. doi: 10.4012/dmj.2018-385. Epub 2019 Oct 2. PMID: 31582598.	Different type of analyze

Table S3. Joanna Briggs Institute Clinical Appraisal Checklist for Experimental Studies.

SELECTION	1	2	3	4	5	6	7	8	9	10	11	OVERALL
Pancu et al. 2011	1	0	0	0	1	1	1	1	1	1	1	8
Meyer-Lueckel et al. 2011	1	0	0	0	1	1	1	1	1	1	1	8
Paris et al. 2011 A	1	0	1	0	1	1	1	1	1	1	1	9
Paris et al. 2011 B	1	0	0	0	1	1	1	1	1	1	1	8
Taher et al. 2012	1	0	0	0	1	1	1	1	1	1	1	8
Torres et al. 2012	1	0	0	0	1	1	1	1	1	1	1	8
Attin et al. 2012	1	0	1	0	1	1	0	1	1	1	1	8
Ekizer et al. 2012	1	1	0	1	1	1	1	0	1	1	1	9
Paris et al. 2013	1	0	1	0	1	1	1	0	1	1	1	8
Paris et al. 2013	1	0	1	0	1	1	1	1	1	1	1	9
Mohammed et al. 2014	1	0	1	0	1	1	1	1	1	1	1	9
Veli et al. 2014	1	1	1	0	1	1	1	1	1	1	1	10
Paris et al. 2014	1	0	1	0	1	1	1	0	1	1	1	8
Lausch et al. 2014	1	0	1	0	1	1	1	1	1	1	1	9
Gelani et al. 2014	1	0	0	0	1	1	1	1	1	1	1	8
Dilber et al. 2014	1	1	1	0	1	1	1	0	1	1	1	9
Arslan et al. 2015	1	0	1	0	1	1	1	1	1	1	1	9
Montasser et al. 2015	1	0	1	0	1	1	1	1	0	1	1	8
Min et al. 2015	1	0	0	0	1	1	1	1	1	1	1	8
Vianna et al. 2015	1	1	1	0	1	1	1	1	1	1	1	10
Baka et al. 2016	1	1	1	0	1	1	1	1	1	1	1	10
Gurdigan et al. 2016	1	0	1	0	1	1	1	0	1	1	1	8
Abdel-Hakim et al. 2016	1	0	1	0	1	1	1	1	1	1	1	9
El-zankalouny et al. 2016	1	0	1	0	1	1	1	1	1	1	1	9
Abdellatif et al. 2016	1	0	0	0	1	1	1	1	1	1	1	9
Neto et al. 2016	1	0	1	0	1	1	1	0	1	1	1	8
Horuztepe et al. 2017	1	0	1	0	1	1	1	1	1	1	1	9
Mandava et al. 2017	1	0	1	0	1	1	1	1	1	1	1	9
Aziznezhad et al. 2017	1	0	1	0	1	1	1	1	1	1	1	9
Prajapati et al. 2017	1	1	0	0	1	1	1	1	1	1	1	8
Rosianu et al. 2017	1	0	1	0	1	1	0	1	1	1	1	8
Yazkan et al. 2018	1	0	1	0	1	1	1	1	1	1	1	9
Khalid et al. 2018	1	0	1	0	1	1	1	1	1	1	1	9
Enan et al. 2018	1	0	1	0	1	1	1	1	1	1	1	9
Nabil et al. 2018	1	0	0	0	1	1	0	1	1	1	1	7
Attia et al. 2018	1	0	0	0	1	1	1	1	1	1	1	8
Askar et al. 2018	1	0	1	0	1	1	1	0	1	1	1	8
Enan et al. 2019	1	0	1	0	1	1	1	0	1	1	1	8
Aswani et al. 2019	1	0	0	0	1	1	1	1	1	1	1	8
Arora et al. 2019	1	0	0	0	1	1	1	1	1	1	1	8
Theodory et al. 2019	1	0	1	0	1	1	1	0	1	1	1	8
López et al. 2019	1	0	1	0	1	1	1	1	1	1	1	9
Gulec et al. 2019	1	1	1	0	1	1	0	1	1	1	1	9
Borges et al. 2019	1	0	0	0	1	1	1	1	1	1	1	8
Ayad et al. 2020	1	0	1	0	1	1	1	1	1	1	1	9
Behrouzi P et al. 2020	1	0	1	0	1	1	1	1	1	1	1	9
El Meligy, 2020	1	0	1	0	1	1	1	1	1	1	1	9
Wang et al. 2020	1	0	1	0	1	1	1	1	1	1	1	9

1. Was the aim of the study clearly stated? 2. Was the sample size justified? 3. Was the assignment to treatment groups truly random? 4. Were those assessing the outcomes blind to the treatment allocation? 5. Were control and treatment

groups comparable at entry? 6. Were groups treated identically other than for the named interventions? 7. Was the preparation protocol clearly described? 8. Was the experimental protocol clearly described? 9. Were outcomes measured in the same way for all groups? 10. Were outcomes measured in a reliable way? 11. Was appropriate statistical analysis used?

Table S4. Comparison between SMD and ROM results.

Variable	N	SMD	95% CI	I^2 (%)	N	ROM	95% CI	I^2 (%)
**Surface Roughness**								
Sound enamel	5	−7.78	−13.22; −2.33	98.1	5	0.65	0.49; 0.85	98.2
WSL	8	−2.43	−3.01; −1.67	95.2	8	0.46	0.29; 0.74	98.5
**Enamel Microhardness**								
Sound enamel	14	−3.35	−4.80; −1.91	97.4	14	0.76	0.73; 0.8	99.1
WSL	23	4.08	2.76; 5.41	96.6	23	1.68	1.51;1.86	99.8
**Bond Strength**								
Sound enamel	6	-0.89	−1.92; 0.14	92.2	6	0.75	0.60; 0.95	96.9
WSL	8	3.40	1.33; 5.48	97.1	8	1.89	1.28; 2.79	99.8

AgP—Aggressive Periodontitis; CP—Chronic Periodontitis; CRP—C-reactive protein; H—Healthy periodontium; hs-CRP—high sensitivity CRP; ROM—Ratio of Mean; SMD—Standardized Mean Difference.

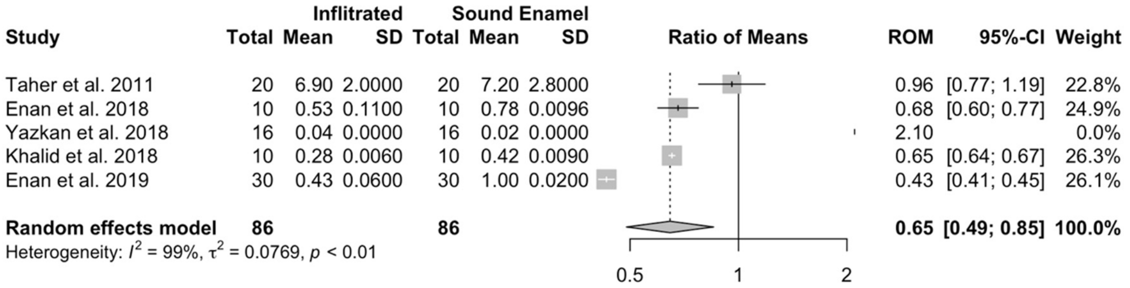

Figure S1. Forest plot of studies evaluating the effect of resin infiltration on the enamel surface roughness of sound enamel. Ratio of means have been calculated with 95% confidence intervals and are shown in the figure. Area of squares represents sample size, continuous horizontal lines and diamonds width represents 95% confidence intervals. Diamond and the vertical dotted line represent the overall pooled estimate.

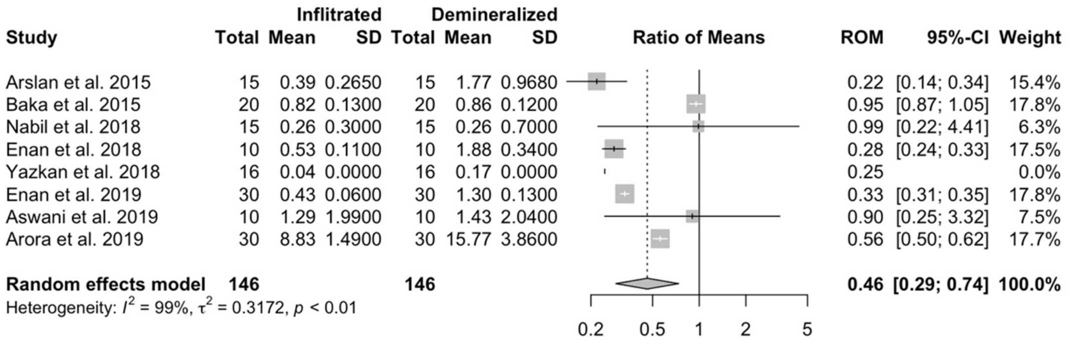

Figure S2. Forest plot of studies evaluating the effect of resin infiltration on the enamel surface roughness (Ra) of white spot lesions. Ratio of means have been calculated with 95% confidence intervals and are shown in the figure. Area of

squares represents sample size, continuous horizontal lines and diamonds width represents 95% confidence intervals. Diamond and the vertical dotted line represent the overall pooled estimate.

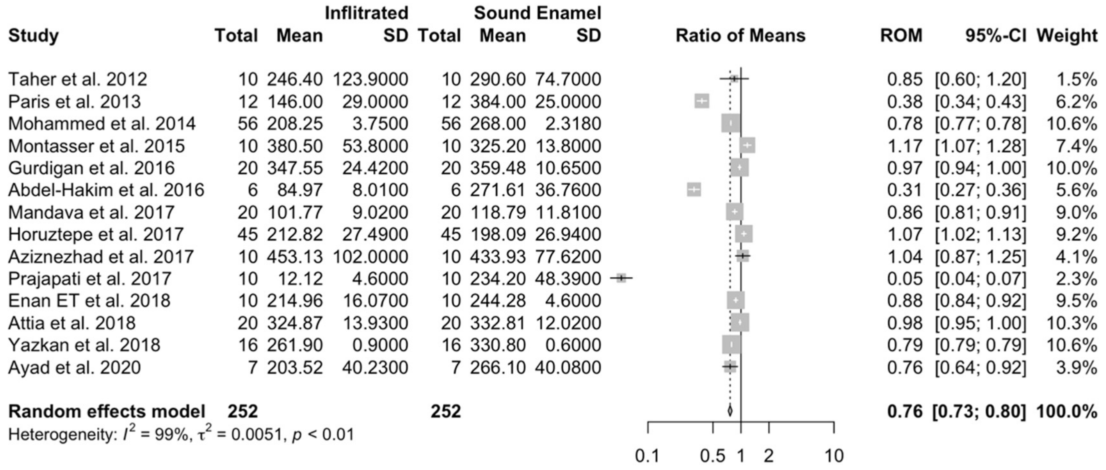

Figure S3. Forest plot of studies evaluating the effect of resin infiltration on the enamel microhardness of sound enamel. Ratio of means have been calculated with 95% confidence intervals and are shown in the figure. Area of squares represents sample size, continuous horizontal lines and diamonds width represents 95% confidence intervals. Diamond and the vertical dotted line represent the overall pooled estimate.

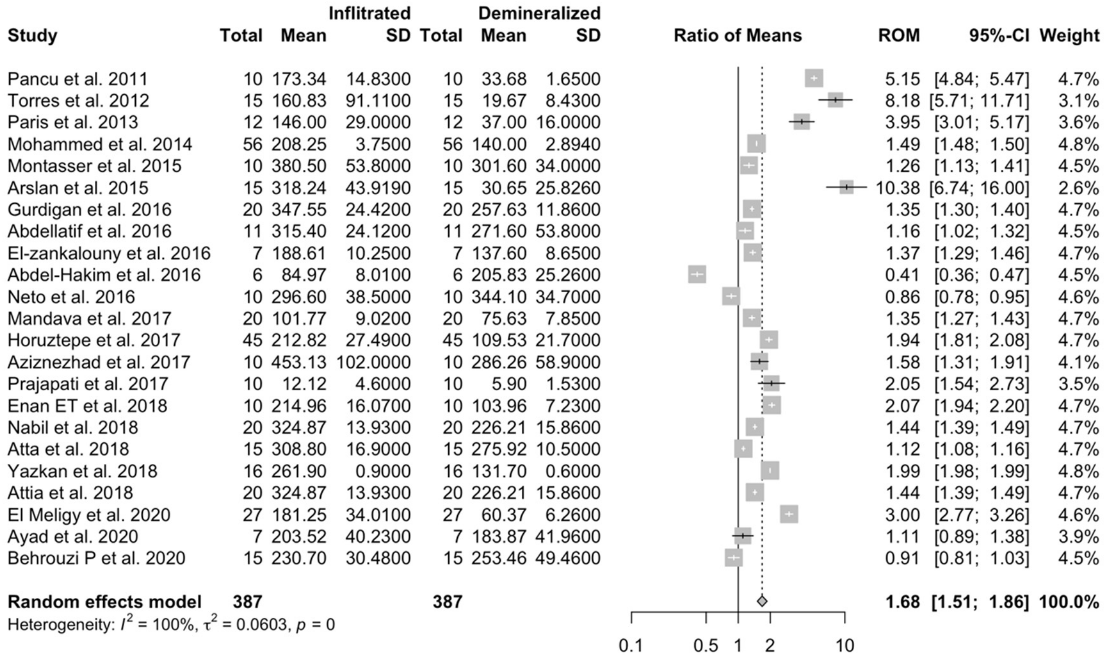

Figure S4. Forest plot of studies evaluating the effect of resin infiltration on the enamel microhardness of white spot lesions. Ratio of means have been calculated with 95% confidence intervals and are shown in the figure. Area of squares represents sample size, continuous horizontal lines and diamonds width represents 95% confidence intervals. Diamond and the vertical dotted line represent the overall pooled estimate.

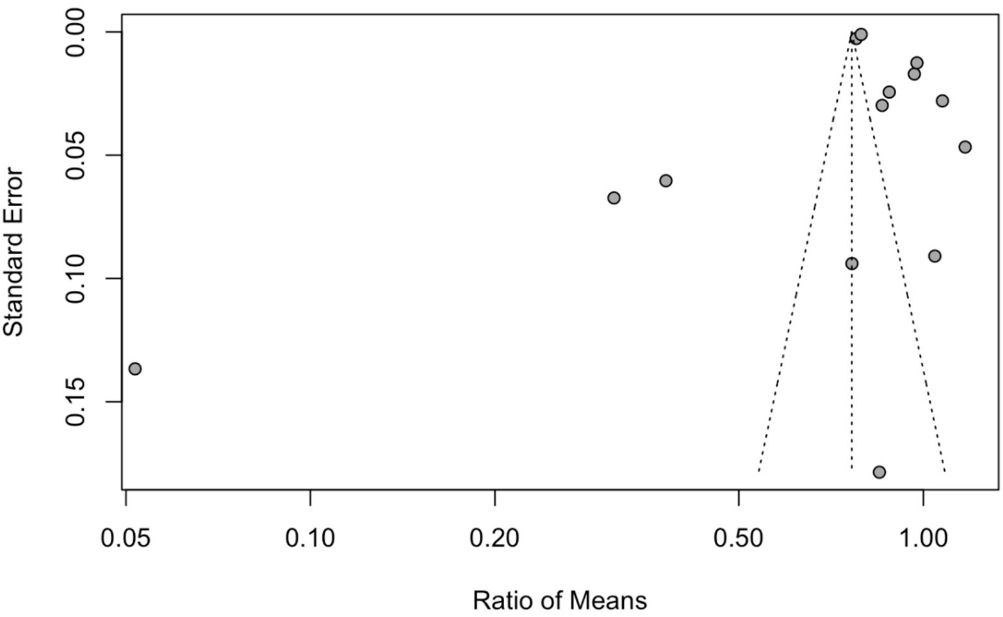

Figure S5. Funnel plot and Egger's test for enamel microhardness in sound enamel.

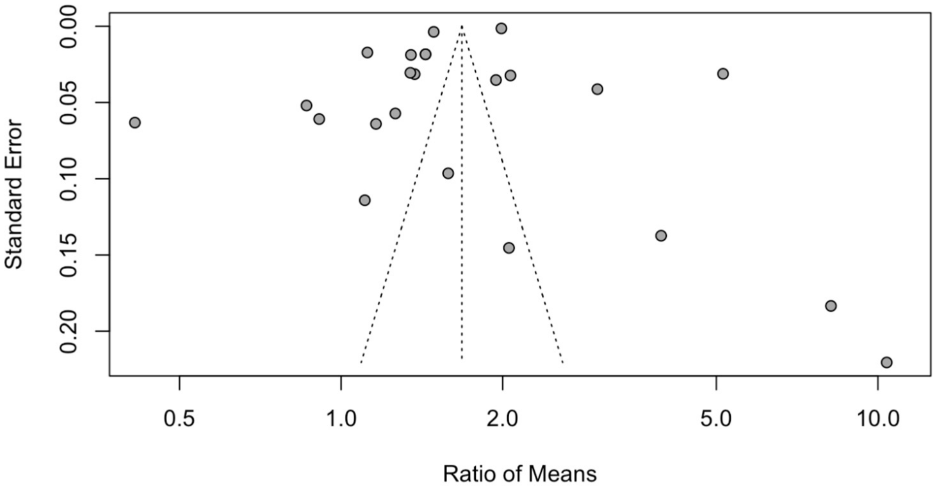

Figure S6. Funnel plot and Egger's test for enamel microhardness in white spot lesions.

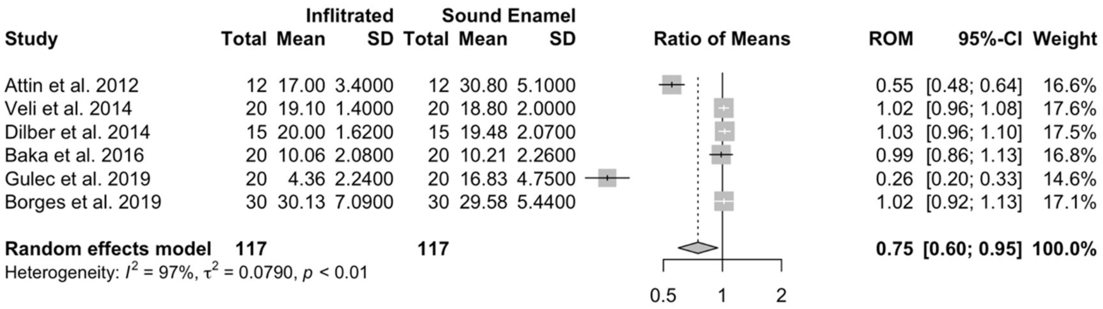

Figure S7. Forest plot of studies evaluating the effect of resin infiltration on the bond strength of sound enamel. Ratio of means have been calculated with 95% confidence intervals and are shown in the figure. Area of squares represents sample

size, continuous horizontal lines and diamonds width represents 95% confidence intervals. Diamond and the vertical dotted line represent the overall pooled estimate.

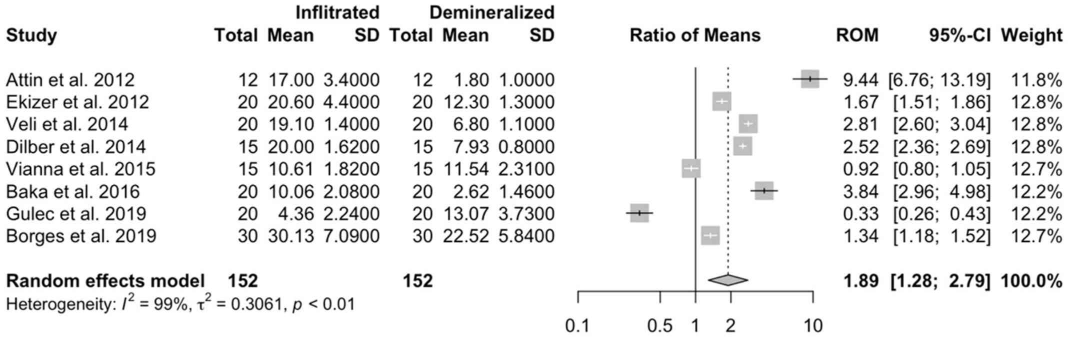

Figure S8. Forest plot of studies evaluating the effect of resin infiltration on the bond strength of white spot lesions. Ratio of means have been calculated with 95% confidence intervals and are shown in the figure. Area of squares represents sample size, continuous horizontal lines and diamonds width represents 95% confidence intervals. Diamond and the vertical dotted line represent the overall pooled estimate.

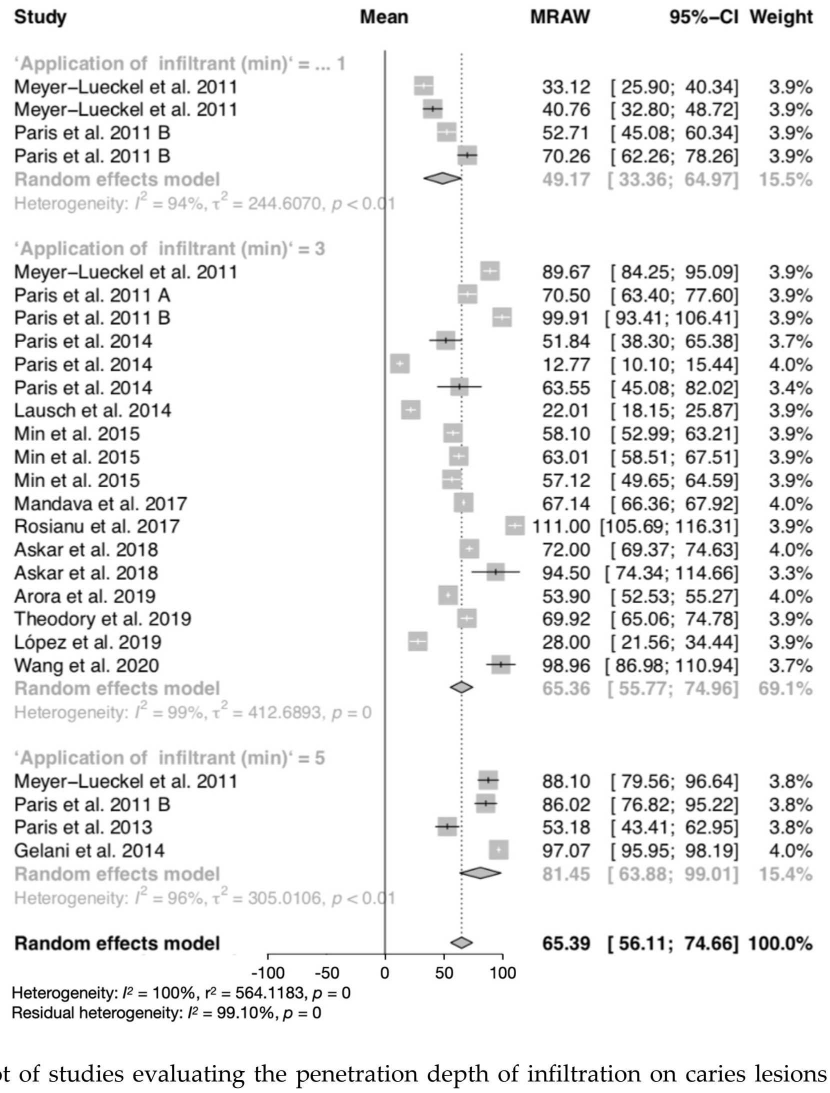

Figure S9. Forest plot of studies evaluating the penetration depth of infiltration on caries lesions according to time of application of infiltrant (in minutes). Ratio of means have been calculated with 95% confidence intervals and are shown in the figure. Area of squares represents sample size, continuous horizontal lines and diamonds width represents 95% confidence intervals. Diamond and the vertical dotted line represent the overall pooled estimate. (z-value < 0.0001); (Egger test = 0.4712)

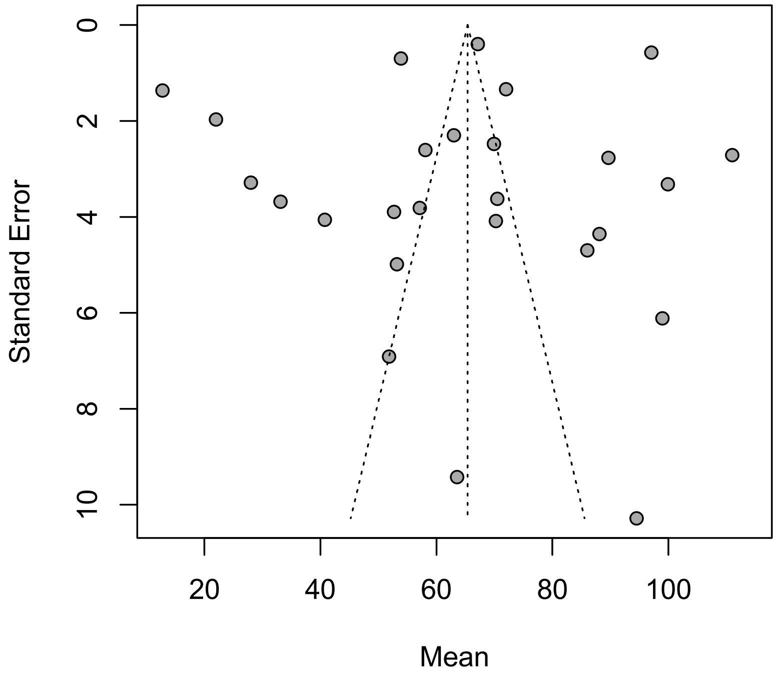

Figure S10. Funnel plot and Egger's test for penetration depth.
